# Experience of Kapandji technique in treating Colles’ fracture in central region of Vietnam

**DOI:** 10.1051/sicotj/2022040

**Published:** 2022-09-28

**Authors:** Le Hoang Nam Dang, Ba Luu Nguyen, Hong Phuc Le, Thanh Thao Nguyen, Nghi Thanh Nhan Le

**Affiliations:** 1 Department of Anatomy-Surgical Training, University of Medicine and Pharmacy, Hue University Hue Vietnam; 2 Department of Surgery, University of Medicine and Pharmacy, Hue University Hue Vietnam; 3 Department of Radiology, University of Medicine and Pharmacy, Hue University Hue Vietnam

**Keywords:** Colles’ fracture, Kapandji technique, Distal radius fractures, K-wire fixation

## Abstract

*Introduction*: Extra-articular fractures of the distal radius, known as Colles’ fractures, are very common. The optimal management of Colles’ fracture is still controversial. The Kapandji technique is one option for orthopedic surgeons to maintain reduced fractures, however, the effectiveness of this method is no clear consensus. This study aims to access Colles’ fracture treatment by the Kapandji technique with our experiences. *Methods:* This prospective study of 33 patients treated with three K-wires intra-focal fractures by the Kapandji procedure for Colles’ fractures at Hue University of Medicine and Pharmacy Hospital in Vietnam between February 2017 and May 2019. The functional outcome of the patients was assessed by the demerit score system of Gartland and Werley, and the quality of reduction was elevated on radiographic as well at 3, 6, 12, and 24 weeks postoperative. *Results:* 33 patients’ mean age is 54.64 ± 18.00; After 24 weeks of postoperative follow-up, 78.79% presented excellent, 21.21% good, and there are not any fair or poor cases on the functional outcome. All patients achieved complete fracture union at 12 weeks postoperative. The average immediate postoperative radial length was 9.85 mm, the radial inclination was 20.64°, and the volar tilt was 9.2°. *Conclusion:* The study emphasizes that the Kapandji technique in Colles’ fracture treatment is simple and possible to bring a satisfactory outcome and fast recovery.

## Introduction

Colles’ fracture is an extra-articular distal radius fracture with the following characteristics: (1) transverse radius fracture; (2) 2.5 cm (0.98″) proximal to the radiocarpal joint; (3) dorsal displacement and dorsal angulation, as well as the radial tilt [1]. Surgical treatment for distal radius fractures aims to restore hand and wrist function early by restoring articular surface congruity and alignment.

Nonoperative therapy is usually recommended for Colles’ fractures. Cast immobilization avoids surgery and complications, but the cast on maintaining distraction to repair the distal radius anatomy has a high failure rate. On the other hand, the predominant indication for plating treatment is distal radius fractures with actual or predicted instability. This approach has been linked to a couple of issues, most of which are hardware-related. Tendon rupture or irritation and screw penetration into the radiocarpal joint are the most common hardware-related problems, resulting in high operation rates [2, 3].

Kapandji described the intrafocal pinning technique for distal radius fractures for the first time in 1976, and it is a simple and successful treatment. Although commonly utilized in Europe, the procedure is still relatively obscure in the United States and Asia. According to the research, there is an ongoing debate about the Kapandji method’s efficacy. In contrast, some authors demonstrated superior anatomical with maintaining reduction and functional outcomes in the group treated with the Kapandji technique, contradicting the findings of several cohort studies that percutaneous pinning only provided a slight improvement in the radiological and did not correlate with an improved functional outcome [4–6].

This study aimed to evaluate the functional and radiological results of Colles’ fractures treated with the Kapandji technique, thereby reinforcing the effectiveness of Kapandji’s technique with our experiences in the central region of Vietnam.

## Materials and method

This is a prospective study of 33 patients (5 males and 28 females, mean age: 54.64 years old) who received the Kapandji procedure for Colles’ fractures at the Hue University of Medicine and Pharmacy Hospital in Vietnam between February 2017 and May 2019.

We excluded elderly patients who presented with severe medical comorbidity, severe comminuted osteoporotic fracture (AO23-C3), and cases of patients delayed >14 days, open fractures, complicated distal radius (stage II, III, IV of Fernandez classification), and surgical contraindications.

### Surgical approach

#### Preparation

T-handle driller, 2.0 K-wire, C-arm. The operations were performed under axillary block or general anesthesia. The approach can be divided into 2 steps:

Step 1: Image intensifier with closed reduction

To restore the radius’s height, using traction and counter-traction during the fluoroscopy. The assistant pulls the thumb of the injured hand along the radial axis while holding the patient’s rest fingers towards the ulna with the other hand. The surgeon presses firmly on the distal fragment inferiorly and towards the ulna. Maintain the wrist in a palmar flexion and ulnar inclination position.

Once again, confirm length restoration, distal radius tilt, and posterior displacement on the image intensifier. The places for placing the K-wires were marked out after the reduction maneuver.

Step 2: K-wire insertion

According to Kapandji’s description, the K-wire is inserted as a lever through the fracture line. Three pins were inserted through the fracture site.

- First pins – lateral side: the intersection of the fracture line with the extensor pollicis brevis and extensor radialis carpi longus tendon intervals.
- Second pins – posterolateral side: the intersection of the fracture line and the extensor pollicis longus-extensor indices tendon interval.
- Third pins – posteromedial side: the intersection of the fracture line and the extensor 4th and 5th finger tendons’ interval.

#### Insertion technique

Incise 5–10 mm at the insertion sites, bluntly dissect soft tissues; put the pin between the dissector, via and through the fracture line, and drill a 45° angle with the radial axis until it penetrates the opposite cortex.

**Figure 1:**
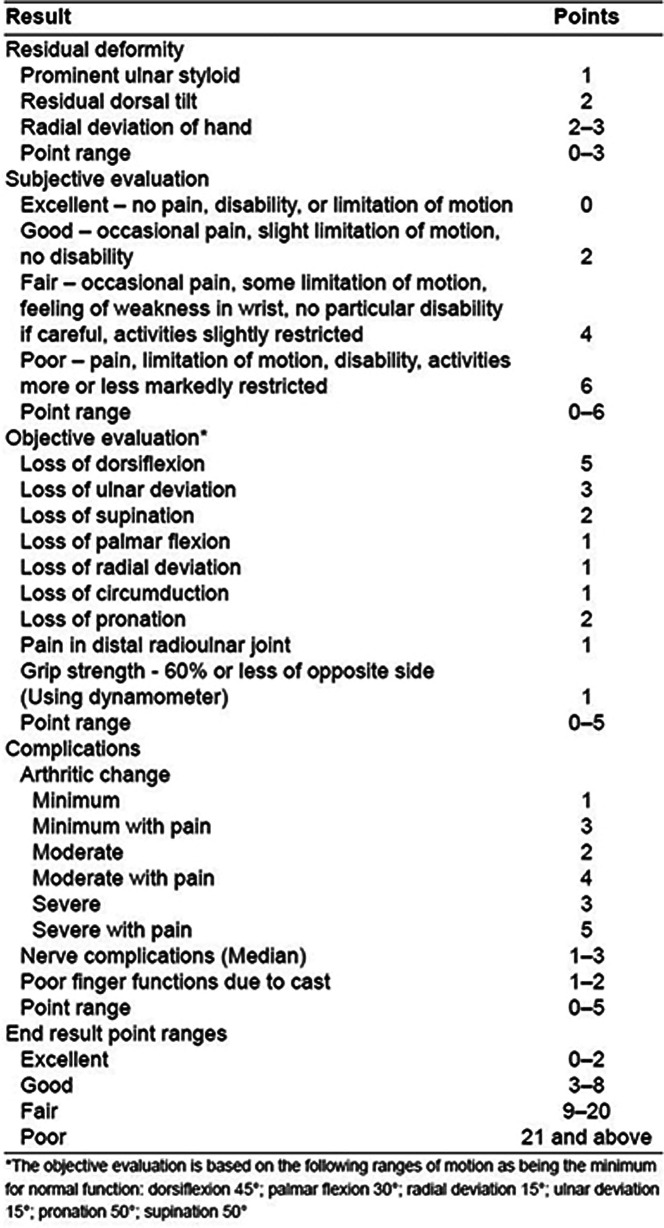
Intraoperative surgical technique: blunt dissect soft tissues and K-wire insertion (a), Fluoroscopic images confirmed the reduction and K-wire position, (b) and X-ray images confirmed bone union at 6 weeks postoperative (c).

Fluoroscopic control was utilized to ensure that the K-wires were placed correctly and that the alignment was satisfactory (Figure 1).

Postoperative (postOP), all patients were applied with the wrist plaster splint in the first 3 weeks. Early finger and wrist motion and rehabilitation were encouraged, as could be tolerated. The pin removal at 6 weeks postOP.

### Clinical outcomes assessment

**Figure 2:**
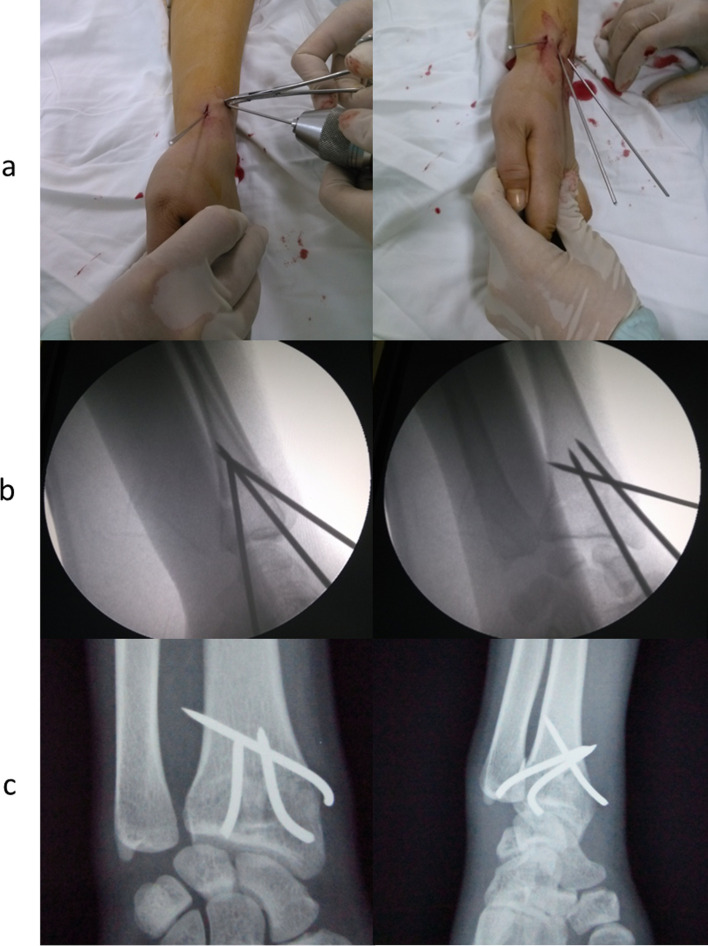
The Garland & Werley scoring system.

Clinical outcomes were evaluated at 3, 6, 12, and 24 weeks postOP according to the Gartland and Werley scoring system, a validated physician-based scoring system, which combines residual deformity, subjective findings, ROM, postoperative complications, and poor finger function. The scale ranges from 0 to 52, with lower values indicating better function and higher levels indicating the worst function (Figure 2).

Any complication, such as infection, K-wire loosening, neuropathy or nerve injury, tendon injury or tendinopathy, chronic regional pain syndrome, malunion, nonunion, re-fracture, and others, was documented at each visit (postOP: 3, 6, 12, and 24 weeks), either from patients’ self-reports or doctors’ check-ups.

### Radiographic assessment

The quality of the reduction was evaluated at 3, 6, 12, and 24 weeks postOP. The parameters of radial inclination, radial length, and volar tilt were measured using standard anterior-posterior and lateral radiography. We used the postoperative radiographic parameters established by Scheck to evaluate the results (Table 1) [7].

**Table 1:** Radiographic assessment by Scheck System.

Scheck classification	Radial length (mm)	Radial inclination (°)	Volar tilt (°)
Excellent	10–13	18–23	6–11
Good	5–9	10–17	0–5
Fair	<5	<10	<0

### Statistical analysis

A medical statistician conducted a power analysis, determining the sample size required to detect any significant change in radial length or dorsal inclination and a difference in Gartland and Werley scores and Scheck. Because of the non-normal distribution, data were summarized using medians rather than means. For the same reason, non-parametric Mann–Whitney *U*-tests were used to comparing groups. SPSS version 19.0 was applied in this study.

## Results

A total of 33 (male: 5 [15.15%]; female: 28 [84.85%]) patients were investigated in our study. The patient’s age averaged 54.64 years. Timing to surgery averaged 7.33 h, and the operation time averaged 34.97 min. Patient demographics are presented in Table 2.

**Table 2:** Demographic data of 33 patients.

Patient	*N* (%)
Age (y)	
Mean ± *SD*	54.64 ± 18.00
Median (min, max)	62 (20.88)
Injured side	
Right	15 (45.40)
Left	18 (54.60)
Sex	
Male	5 (14.50)
Female	28 (85.50)
Time to surgery (h)	
Mean ± *SD*	7.33 ± 4.34
Median (min, max)	7 (4.25)
Operative time (min)	
Mean ± *SD*	34.97 ± 13.00
Median (min, max)	40 (20.90)

### Clinical outcomes assessment

According to the Gartland and Werley scoring system, good to excellent outcomes could be achieved in all cases; there were no fair and poor results, as depicted in Table 3. Overall, 24 of 33 (72.7%) and 26 of 33 (78.79%) patients had an excellent outcome at 12 and 24 weeks postOP, respectively.

**Table 3:** Clinical outcomes by Gartland & Werley Score

Follow-up period (Weeks)	Excellent		Good	
	*N*	%	*N*	%
3	25	75.80	8	24.20
6	24	72.70	9	27.30
12	24	72.70	9	27.30
24	26	78.79	7	21.21

### Radiographic assessment

All patients achieved complete fracture union at 12 weeks postOP. The average immediate postoperative radial length was 9.85 mm, the radial inclination was 20.64°, and the volar tilt was 9.2°. Statistically significant differences were found between the preOP and postOP parameters (Table 4). According to the Scheck score system, the results of X-ray outcomes were achieved from good to excellent in all cases, as depicted in Table 5.

**Table 4:** Comparison of Pre OP and Post OP parameters

Measurement	Pre OP (Mean ± sd)	Post OP (Mean ± sd)	*p*
Radial inclination (°)	15.24 ± 3.58	20.64 ± 2.39	<0.01
Radial length (mm)	4.88 ± 3.18	9.85 ± 1.62	<0.01
Volar tilt (°)	2.21 ± 5.61	9.24 ± 2.10	<0.01

**Table 5:** X-ray outcomes by Scheck Score

Follow-up period (Weeks)	Excellent		Good	
	*N*	%	*N*	%
3	24	72.70	9	27.30
6	24	72.70	9	27.30
12	22	66.67	11	33.33
24	25	75.80	8	24.20

### Complications

No major medical complications were recorded during surgery. There was no infection, delayed union, or nonunion in any of the patients. One patient had presented radial nerve injury postoperatively with only numbness at the index finger. However, after intensive conservative treatment, the symptom completely disappeared after 12 weeks.

## Discussion

Despite being the most frequent upper extremity fracture, Colles’ fractures continue to provide a therapeutic difficulty. The goal is to regain a normal range of motion and anatomical integrity while avoiding pain. These fractures can be treated and stabilized in several ways. These include conservative management with cast immobilization and surgical methods such as percutaneous pinning, internal fixation, and external fixation [8]. Since its description in 1976, Kapandji’s technique of intra-focal pinning has been the preferred operation technique in Colles’ fracture in France and other European countries. This is a simple and less invasive method but also maintains reduction and function outcomes [9]. In contrast, some research is still suspicious concerning the potential loss of anatomical restoration and its impact on function [4].

In our research, the Kapandji technique has been shown to improve both anatomical and functional outcomes. At 6 months postOP, 78.79% of patients had achieved excellent and 21.21% good results; there were no cases with fair and poor results. Regarding radiography results, the postoperative radial length was 9.85 ± 1.62 mm, which is near to normal radius length (the normal value is around 1 mm) – a good result. The radial inclination was 20.64 ± 2.39°, while the volar tilt increased from 2.21 ± 5.61° PreOP to 9.24 ± 2.10° PostOP. They were all approximate to their normal value (15–25°) with the radial inclination and 11–12° with dorsal tilt [10]. These results demonstrated that this method also successfully maintains radial length, dorsal angulation reduction, and radial inclination.

Although the Kapandji technique is a simple process, it still has some complications. After surgery, one patient was found to have nerve injury. The superficial branch of the radial nerve is usually injured, resulting in numbness along the dorsal skin between the index and middle fingers. In this situation, the patient displayed identical symptoms. However, the numbness was gone after 12 weeks of intense therapy with conservative treatment. After making a skin incision, dissect the soft tissue until it reaches the bone membrane, then implant the K-wire beyond the bone to avoid this problem. This technique also minimizes the incidence of tendon tears. Most surgeons worry about superficial wound infection, one reason they prefer plate to percutaneous K-wire fixation. Fortunately, no infections were reported in our patients, causing our number to be small, and the tip of the K-wire was bent and hidden under the skin. On the other hand, the theatre time and implant cost of percutaneous K-wire fixation are lower than the plate osteosynthesis, so this is a good option for poor and developing countries [11].

There are several limitations to the current study. First, compared to previous research, our sample size was small, and the follow-up time was short. Long-term outcomes and complications, such as wrist osteoarthritis, should be followed. Second, no additional reduction techniques were compared in our research. Because it lacked a designated primary outcome measure and a formal estimate of sample size, it may be necessary to conduct another comparative research. As a result, the study’s sample size was probably insufficient to discover clinically meaningful differences in functional outcomes and complication rates.

The main disadvantage of the Kapandji technique is that it does not provide enough stability and is challenging to maintain the prior anatomical reduction. The fracture tends to collapse in elderly patients, a common occurrence in the literature [4, 6, 12]. In the study of Barton et al., 10 of 53 (19%) patients with manipulation and longitudinal K-wiring of displaced Colles’ fractures failed to achieve an adequate reduction, especially radial shortening [5]. According to our experience, the best strategy to reduce the risk of this problem includes: firstly, we always perform closed manipulation to achieve good radiography parameters with C-arm before K-wire insertion. The second limitation is the number of times the K-wire insertion prevents breaking or weakening of the opposing cortical bone and loosening the K-wire following surgery. Third, we always use three K-wires (one at lateral, two at posterolateral and posteromedial size) to maintain reduction, while the technique of intra-focal pinning was originally described by Kapandji, who used two pins for unstable extra-articular fractures [9], and finally, all patients in our study were applied with the wrist plaster splint in the first 3 weeks.Patients can safely practice early mobilization with three K wires, even with a splint. As a result, the patient feels more comfortable than the cast and avoids complications from the cast and prolonged immobilization. We did not perform the direct reduction of the fracture with the K-wire because elderly patients at risk of osteoporosis may cause bone subsidence or fracture at the contact site of the K-wire, so it is difficult to achieve the inclination and length of the distal radius. Suppose the patient has a comminuted fracture at the fracture site, the risk of difficulty in restoring the anatomical morphology of the distal radius. In that case, we prefer to proceed with over reduction and stabilize the fracture with K-wire and cast. Some authors have demonstrated that using a combination of Kapndji and external fixation can be suitable in situations like these [13].

Over time, methods for treating Colles’ fracture have changed, and in the past century, several operational techniques for reduction and fixation were developed. However, no matter what method, the goal of treatment is to achieve good functional outcomes requiring good reduction. In the opinion of many orthopedic surgeons, the alignments are essential for predicting functional results. One recent study reported a dorsal tilt of 12° or more, and >2 mm of the radial shortening was associated with significant functional limitation [14]. For the above reasons, some authors doubt the Kapandji method, which is an insufficiency stable fixation and may lead to a poor reduction and late collapse of the fracture after removing the K-wire [15]. Interestingly, according to the data from the literature, the radiographic reduction results in our study were comparable with different methods (Table 6) [16–19]. In fact, open reduction and internal fixation by volar locking plates achieve better anatomical reduction and fixation stability. Biomechanical studies have demonstrated that volar locking plates are considerably more stable than percutaneous pins in unstable distal radius fractures [20, 21]. However, for Colles’ fracture, an unsatisfactory radiological outcome does not necessarily result in an unsatisfactory functional outcome, especially in elderly patients. Some loss of reduction can be expected due to osteopenia and comminution [6]. On the other hand, the consolidation of the distal radial fracture is very quick, so after 3 weeks, the patients treated with the ORIF or the Kapandji method were rehabilitated under almost the same plan. The patients treated by the Kapandji method have less pain due to small incisions and avoid complications of open surgery, so patients will practice early more easily. In general, factors that must be considered while using a volar locking plate are the risk-benefit analysis according to the specific complications of each treatment option and operative technique, the patient’s functional condition and demand, and the cost-effectiveness of the treatment option. Most review articles show small differences in the results between the two techniques and are unlikely to be clinically important [16, 19, 22–24].

**Table 6:** Comparison data from the literature.

Measurement PostOP	Our study	Chung et al. [16]	Kennedy et al. [17]	Saddiki et al. [19]		Jirangkul et al. [20]
	Kapandji	VLP	K-wire & Cast	Kapandji	PY’s	LCP 3.5
Radial inclination (°)	20.64	24	21.6	22.5	25.57	21.15
Radial length (mm)	9.85	12	≥ −2	9	9.9	10.89
Volar tilt (°)	9.24	9	≤15	6.8	10.27	10.23

VLP: Volar locking plate, LCP: Locking compression plate, PY’s: Isoelastic pinning technique.

Despite the loss of reduction, several authors report positive long-term functional outcomes in patients treated with closed reduction and cast immobilization. This is a good option, but it is trending to decrease now because cast immobilization is a limited form of stabilization; they easily lose position, resulting in malunion. Furthermore, several problems associated with long-term immobilization, such as stiffness, arthrosis, median neuropathy, Sudek syndrome, or shoulder-hand syndrome, have been observed with cast immobilization [8, 17, 25]. According to Board et al.’s comparison research of the Kapandji technique and cast immobilization in the treatment of Colles’ fracture, the Kapandji approach is substantially less painful than manipulation and plaster immobilization [6]. Strohm et al. found the functional and radiographic outcomes of the Kapandji method adopted and proved that they are considerably superior to the conventional technique in a prospective randomized study of over 100 distal radius fractures in adults treated with Sommer pins [26].

Several authors investigated applying external fixation to the treatment of Colles’ fracture; although the loss of radius length can be restored with external fixation, however, correction of the palmar tilt and radial inclination is exceedingly challenging without causing distorting to the distal radioulnar joint [27, 28]. Therefore, some authors combined the Kapandji technique with external fixation to provide good reduction and obtained promising results [13].

## Conclusion

Various treatment options for Colles fractures result in an adequate anatomical restoration and acceptable function recovery. The Kapandji approach, on the other hand, seems to be a safe, simple, and low-cost method for treating Colles’ fractures in elderly patients, according to this study. This technique for reduction is not only simple to use, but it is also reliable. In elderly patients with osteopenia, some decreased loss is expected, however, this is considerably better minimized by utilizing the Kapandji approach than manipulation and cast immobilization, as well as avoiding risks associated with long-term cast immobilization. When these techniques are being used, the functional outcome following injury appears to be highly related to the anatomical result.

## Conflict of interest

The authors declare they have no relevant financial or non-financial interests to report.

## Funding

This research did not receive any specific funding.

## Ethical approval

This study received ethical approval (number: H2022/030) from The Institutional Ethics Committee of Hue University of Medicine and Pharmacy, Vietnam.

## Informed consent

Written and informed consent was obtained from every individual.

## Authors contributions

*LHN-D*: Wrote the paper. *BL-N* and *HP-L*: Analyzed the data. *TT-N*: Contributed analysis tools. *NTN-L*: Conceived and designed the study. All authors read and approved the final paper.
